# Supplementary material for: A Systematic Approach to Provide Feedback to Presenters at Virtual and Face-to-Face Professional Meetings
Source: MedEdPORTAL. 2022 Dec 16;18:11288. doi: 10.15766/mep_2374-8265.11288 (PMC9755373; doi:10.15766/mep_2374-8265.11288)
Supplement: Supplementary file 1 — Meeting Organizer Checklist.docxEmail to Presenters (Before Conference).docxSummative Assessment Forms.docFormative Assessment Form.docxEmail to Assessors (Before Conference).docxEmail to Presenters (After Conference).docxEmail to Assessors (After Conference).docxFocus Group Guides.docx [file mep_2374-8265.11288-s001.zip › A. Meeting Organizer Checklist.docx]

**Meeting Organizer Checklist**

**One to Two Months Before the Event**

Generate a list of the complete names and email addresses of presenters selected to give an oral presentation at the event.

Send an email to each presenter prior to the event offering guidance on how to prepare an effective oral presentation (Appendix B) and include examples of the blank summative and formative assessment forms (Appendices C and D).

Recruit assessors with expertise in medical education and/or educational scholarship who plan to attend the event. In recruiting assessors, be mindful of demographic diversity and strive to ensure that your assessors represent a wide range of stakeholders in educational scholarship. After identifying a pool of assessors, send an individual email to each assessor asking them to specify their preference to provide formative or summative feedback. In this email, be sure to include blank versions of the formative and summative assessment forms to clarify expectations (Appendices C and D).

**Two Weeks Before the Event**

Develop a schedule where two summative assessors and one formative assessor is assigned to provide feedback for all presentations in a given session (usually 3-5 presentations). Pay attention to potential conflicts of interest (i.e., summative assessors are not from the same institution as the presenters), diversity of raters (i.e., expertise areas, degrees, race/ethnicity, gender, etc.), and scheduling constraints (i.e., assessor is giving a presentation at the same time of the assigned session). If feasible, distribute the meeting program to assessors in advance and ask them to specify the session for which they would prefer to provide feedback.

Recruit session moderators, if feasible, who can introduce speakers and solicit questions/monitor chat if virtual meeting rather than assigning those tasks to assessors.

Distribute to assessors (and session moderators, if applicable) an email containing information on their session assignment, links to the electronic assessment forms, and the abstracts for the presentations comprising their assigned session (Appendix E). Ideally, the meeting organizer will pre-populate the assessment forms with the assigned presentation titles and speaker names to make it easier for assessors to document their impressions.

One Week Before the Event

Send a reminder email to the assessors and moderators confirming their assignments and availability.

During the Event

If a face-to-face meeting, verify that moderators and assessors show up to the correct location of their assigned session. If a virtual meeting, have someone who is qualified to serve as an assessor or moderator on standby if an assessor or moderator experiences technical problems and cannot access the assigned session.

Monitor that assessments are getting completed and submitted on a timely basis.

**One Week After the Event**

As needed, send email reminders to assessors who have not submitted their reviews by the deadline of one day after the event.

Approximately 2-3 days after the event, compile the summative assessment forms, and aggregate the ratings on each (e.g., by summing or averaging). This will generate an overall summative score for each presenter. Ideally, the meeting organizer should adopt an aggregation method that is defensible if a presenter has questions.

Add the overall summative score for each presentation to a spreadsheet containing all of the presentations delivered at the event. Then, rank the presentations from highest to lowest based on this score. Select the highest-ranked presentation as the recipient of the Best Oral Presentation award. If overall summative scores lack variability or in the event of a tie, it can be helpful to review comments on the summative assessment forms to make a discrimination.

Summarize the formative and summative assessment form data for each presenter, saving it as a PDF to distribute to each presenter.

Send a personalized email (Appendix F) to each presenter that (1) announces the recipient of the Best Oral Presentation award; and (2) includes the summarized feedback document as an attachment.

Send an email to each group of assessors (summative and formative) assigned to a given session. This email should (1) announce recipient of Best Oral Presentation award; (2) include the summarized feedback given to presenters in the assigned session; (3) present your observations of the feedback process; and (4) solicit feedback about the feedback process (Appendix G).
