# Supplementary material for: A Systematic Approach to Provide Feedback to Presenters at Virtual and Face-to-Face Professional Meetings
Source: MedEdPORTAL. 2022 Dec 16;18:11288. doi: 10.15766/mep_2374-8265.11288 (PMC9755373; doi:10.15766/mep_2374-8265.11288)
Supplement: Supplementary file 1 — Meeting Organizer Checklist.docxEmail to Presenters (Before Conference).docxSummative Assessment Forms.docFormative Assessment Form.docxEmail to Assessors (Before Conference).docxEmail to Presenters (After Conference).docxEmail to Assessors (After Conference).docxFocus Group Guides.docx [file mep_2374-8265.11288-s001.zip › B. Email to Presenters (before conference).docx]

Dear **<Name of presenter>**:

Congratulations on having your abstract accepted for presentation at **<conference name>**. You were selected from a competitive pool of applicants for this honor. Please read the following to ensure a great presentation!

1. In order to keep on time, please limit your slides to a number that is reasonable for a **<number>** minute presentation (approximately one slide per minute or fewer). List any conflicts of interest on your second slide. Please practice your talk so you know that you can fit what you want to say in the time allotted.

2. Be sure that the person presenting your research is familiar with the topic and able to answer questions. Having someone who was a major contributor to the work and understands the content well will ensure a good learning opportunity for the audience.

3. Remember that the audience at **<conference name>** represents a variety of backgrounds. Make sure that the study aims and design are clear to a professionally diverse audience and use [inclusive language](https://www.aamchealthjustice.org/narrative-guide) (<https://www.aamchealthjustice.org/narrative-guide>) that is inviting to people of all demographic and social identities.

4. Though you may be creative in your approach for organizing your presentation, an organization that works well for research presentations is:

1. Background - what does the literature show and what is your study question?
2. Methods - type of study, sample, setting, intervention, analyses
3. Results - demographics of participants/participation rate, major findings
4. Discussion - your findings in context, what your study adds to what is currently known about this topic and next steps

5. Make sure the writing on your slides is readable (no more than 4-6 lines text/slide).

6. Tables and graphs provide an effective way to present main results. Please check that your tables and graphs are easy for the audience to see and interpret.

As in previous years, this year we will provide each presenter with formative and summative feedback within one week following the **<conference name>**. I have attached the assessment forms for you to preview. A combination of presentation ratings and written comments will be used to select a **<name of award>** within 30 days following the **<conference name>**.

I look forward to a great group of presentations. Please contact me with any questions.

Best Regards,

**<meeting/feedback organizer name>**

**<title>**
