# Supplementary material for: A Systematic Approach to Provide Feedback to Presenters at Virtual and Face-to-Face Professional Meetings
Source: MedEdPORTAL. 2022 Dec 16;18:11288. doi: 10.15766/mep_2374-8265.11288 (PMC9755373; doi:10.15766/mep_2374-8265.11288)
Supplement: Supplementary file 1 — Meeting Organizer Checklist.docxEmail to Presenters (Before Conference).docxSummative Assessment Forms.docFormative Assessment Form.docxEmail to Assessors (Before Conference).docxEmail to Presenters (After Conference).docxEmail to Assessors (After Conference).docxFocus Group Guides.docx [file mep_2374-8265.11288-s001.zip › D. Formative Assessment Form.docx]

MESRE Oral Presentation – Formative Feedback

Title of Presentation: No. of Session:

Presenter: Date:

What did you appreciate about this presentation/topic? (Consider introduction, problem/goals, methods, project significance, and inclusivity of framing and language)

What suggestions do you believe would strengthen this project and/or enhance the speaker’s presentation of the topic? (Please comment specifically on any suggestions for enhancing presentation with respect to inclusivity and, if applicable, the virtual environment.)

What “pearls” or “best practices” did you take from this presentation?

What future directions for this project, if any, do you want the presenter to consider?
