# Supplementary material for: A Systematic Approach to Provide Feedback to Presenters at Virtual and Face-to-Face Professional Meetings
Source: MedEdPORTAL. 2022 Dec 16;18:11288. doi: 10.15766/mep_2374-8265.11288 (PMC9755373; doi:10.15766/mep_2374-8265.11288)
Supplement: Supplementary file 1 — Meeting Organizer Checklist.docxEmail to Presenters (Before Conference).docxSummative Assessment Forms.docFormative Assessment Form.docxEmail to Assessors (Before Conference).docxEmail to Presenters (After Conference).docxEmail to Assessors (After Conference).docxFocus Group Guides.docx [file mep_2374-8265.11288-s001.zip › E. Email to Assessors (before conference).docx]

Dear Colleagues,

Thank you for agreeing to provide **<conference name>** presenters with anonymous feedback about their oral presentations.

In addition to a moderator, we will have three assessors for each presentation: two summative raters and one formative assessor.

You are scheduled for **<session name, time, and location>**.

Moderator **<name>**

Summative Rater 1: **<name>**

Summative Rater 2: **<name>**

Formative Assessor: **<name>**

If you are the session moderator, please introduce each speaker and prepare 1-2 questions to ask during the discussion following each presentation. (I asked each presenter to limit their talk to **<N minutes>** to provide time for questions/discussion.) Please also arrive to the session 5-10 minutes early to assist speakers with preparing their presentations.

This year, we will be using online forms to capture summative ratings and formative feedback. The forms are available as follows:

**<hyperlink to summative assessment form>**

**<hyperlink to formative assessment form>**

These forms are in a survey format, with a unique survey for each presentation. Please be sure you have the appropriate link above easily accessible on your laptop, tablet, or mobile device so that you can fill out the form for each presentation during the session. Please use the form to answer each question or rate each item on the assessment form to the best of your ability. The formative assessor may find it easier to fill out the form using a laptop or tablet (vs. phone) to make typing easier. I have attached the abstracts for your assigned presentations if you’d like to review them in advance of the session.

I will collate your anonymous ratings and comments and send them to each presenter following the meeting. The Program Planning Committee will use these data to determine who should receive the “Best Oral Presentation Award” (announced after the meeting). In addition, presenters should be able to use your comments formatively to improve their presentations and advance the scholarly output of their projects.

Thanks again for volunteering to help with the meeting. Please email me if you have any questions. I look forward to seeing you!

All the best,

**<meeting/feedback organizer name>**

**<title>**
