# Supplementary material for: A Systematic Approach to Provide Feedback to Presenters at Virtual and Face-to-Face Professional Meetings
Source: MedEdPORTAL. 2022 Dec 16;18:11288. doi: 10.15766/mep_2374-8265.11288 (PMC9755373; doi:10.15766/mep_2374-8265.11288)
Supplement: Supplementary file 1 — Meeting Organizer Checklist.docxEmail to Presenters (Before Conference).docxSummative Assessment Forms.docFormative Assessment Form.docxEmail to Assessors (Before Conference).docxEmail to Presenters (After Conference).docxEmail to Assessors (After Conference).docxFocus Group Guides.docx [file mep_2374-8265.11288-s001.zip › F. Email to Presenters (after conference).docx]

************************ [PRESENTER’S FEEDBACK EMAIL] ************************

Dear **<Name of presenter>**:

As you know, at **<conference name>** we provide all presenters with formative and summative feedback on their work. Three of your colleagues were dedicated observers of your oral presentation, two providing summative ratings and one providing formative commentary. As promised, I have attached a summary of their feedback for your review.

This year’s Best Oral Presentation award was given to:

**<presentation title>**

***<authors>***

Thank you for contributing to an exceptionally high-quality program at this year’s meeting. Your good work made the decision very difficult this year! I appreciate YOUR feedback on how we can optimize the feedback you received on your presentation or provide you with better guidance on how to prepare for your next one – at **<conference name>** or elsewhere.

Best wishes with your scholarly work!

Kind Regards,

**<meeting/feedback organizer name>**

**<title>**

************************ [BEST PRESENTATION AWARDEE] ************************

Dear **<Name of presenter>**:

As you know, at **<conference name>** we provide all presenters with formative and summative feedback on their work. Three of your colleagues were dedicated observers of your oral presentation, two providing summative ratings and one providing formative commentary.

**This year, as an outcome of our review process, your presentation was selected as the Best Oral Presentation.** Please accept my heartfelt congratulations for a job well done.

As promised, I have also attached a summary of the feedback on your presentation for your review.

Thank you for contributing to an exceptionally high-quality program at this year’s meeting. I appreciate YOUR feedback on how we can optimize the input you received on your presentation or provide you with better guidance on how to prepare for your next one – at **<conference name>** or elsewhere.

Best wishes with your scholarly work!

Kind Regards,

**<meeting/feedback organizer name>**

**<title>**
