# Supplementary material for: A Systematic Approach to Provide Feedback to Presenters at Virtual and Face-to-Face Professional Meetings
Source: MedEdPORTAL. 2022 Dec 16;18:11288. doi: 10.15766/mep_2374-8265.11288 (PMC9755373; doi:10.15766/mep_2374-8265.11288)
Supplement: Supplementary file 1 — Meeting Organizer Checklist.docxEmail to Presenters (Before Conference).docxSummative Assessment Forms.docFormative Assessment Form.docxEmail to Assessors (Before Conference).docxEmail to Presenters (After Conference).docxEmail to Assessors (After Conference).docxFocus Group Guides.docx [file mep_2374-8265.11288-s001.zip › G. Email to Assessors (after conference).docx]

Dear Colleagues,

Thank you so much again for volunteering your service to <**conference name/year>**.

I found the overall quality of the MESRE sessions to be exceptional this year. I believe the presentation support, rating, and feedback process started by Beth Bierer, the previous MESRE Chair, has a lot to do with this. The thoughtfulness of your efforts this year will continue this positive trend!

The presentation receiving this year’s **<title/name of award>** is follows:

**<Title>**

**<Presenter(s)’ names>**

Anticipate a formal announcement of all award winners via **<communication venue/approach>**.

I plan to make the following recommendations about presentation support and judging at the next **<conference oversight group>** meeting:

- **<list recommendations>**

Do you have other recommendations or observations?

Again, I appreciate your commitment to give others feedback on their oral presentations. Thank you for your service!

Best Regards,

**<Name of conference/feedback organizer>**
