# Supplementary material for: A Systematic Approach to Provide Feedback to Presenters at Virtual and Face-to-Face Professional Meetings
Source: MedEdPORTAL. 2022 Dec 16;18:11288. doi: 10.15766/mep_2374-8265.11288 (PMC9755373; doi:10.15766/mep_2374-8265.11288)
Supplement: Supplementary file 1 — Meeting Organizer Checklist.docxEmail to Presenters (Before Conference).docxSummative Assessment Forms.docFormative Assessment Form.docxEmail to Assessors (Before Conference).docxEmail to Presenters (After Conference).docxEmail to Assessors (After Conference).docxFocus Group Guides.docx [file mep_2374-8265.11288-s001.zip › H. Focus Group Guides.docx]

**Structured Guide for Focus Group with Presenters**

**Regarding their Perceptions of Feedback following Regional CGEA Conference**

**Number of Participants:**  **Length of Focus Group (Min:Sec):** :

**INTRODUCTION (Allot 3-5 minutes)**

*Thank you for taking the time to meet with us. Our overall goal during this focus group is to learn how the Medical Education Scholarship, Research and Evaluation section – also known as MESRE – can better support the professional development and careers of health professions educators and scholars, like yourselves. We are especially interested in your perceptions about the guidance and feedback you recently received from MESRE during and after this year’s virtual CGEA meeting. We will use your feedback to improve how we assist those who present their educational scholarship at medical education conferences. I would like to record our discussion. This will let me focus on what you say rather than take a lot of notes. Your name will not appear on the minutes of our discussion or my notes. Please use the poll to let me know if recording this session is okay with you [launch poll – record or don’t record based on vote].*

*As we’ve all become more familiar with Zoom, I ask that you turn your camera on so we can see each other during the conversation. I would appreciate equal participation - through dialogue or Zoom features - as everyone’s experience and opinions matter to us. [Display how to access chat and participation icons in Zoom].*

*Let’s begin with briefly introducing ourselves by giving name, current role, and institution. Then, I’ll ask about your experience at this year’s CGEA spring meeting.*

**PREPARATION FOR/OBSERVATIONS DURING VIRTUAL SESSION** (**Allot 15-20 minutes)**

**1. *What do you wish you would have known ahead of time, if anything, prior to giving your virtual presentation at the CGEA spring meeting?***

**2. *What would have helped you?***

**3. *Did you observe other virtual presentations at the CGEA meeting? If so, what are some of the best practices that stand out in your mind when observing other virtual presentations?***

**4. *How can the MESRE community support the professional development of others?***

**PERCEPTIONS OF FEEDBACK (Allot 15-20 minutes)**

*This year we asked MESRE members to observe virtual presentations and provide each speaker with formative and summative feedback.*

**5. *What were your initial impressions of the feedback you received after your presentation at this year’s CGEA meeting?***

**6. *To what extent did you find this feedback actionable? Please explain.***

**7. *How do you plan to use this feedback going forward?***

**8. *What advice should we share with our peer reviewers when they give feedback to others?***

**OTHER (Allot 5 minutes)**

**9. *What else would you like to tell us about this process that we did not already discuss?***

**Structured Guide for Focus Group with Providers of Formative Feedback**

**After the Regional CGEA Conference**

**Number of Participants:**  **Length of Focus Group (Min:Sec):** :

**INTRODUCTION (Allot 3-5 minutes)**

*Thank you for taking the time to meet with us. Our overall goal during this focus group is to learn how the Medical Education Scholarship, Research and Evaluation section – also known as MESRE – can better support the professional development and careers of health professions educators and scholars. We are especially interested in learning more about your experience while providing presenters with formative feedback at this year’s CGEA meeting. We will use your feedback to improve how we assist those who provide feedback to others who present educational scholarship at medical education conferences. I would like to record our discussion. This will let me focus on what you say rather than take a lot of notes. Your name will not appear on the minutes of our discussion or my notes. Please use the poll to let me know if recording this session is okay with you [launch poll – record or don’t record based on vote].*

*As we’ve all become more familiar with Zoom, I ask that you turn your camera on so we can see each other during the conversation. I would appreciate equal participation - through dialogue or Zoom features - as everyone’s experience and opinions matter to us. [Display how to access chat and participation icons in Zoom].*

*Let’s begin with briefly introducing ourselves by giving our name, current role, and institution. Then, I’ll ask about your experience at this year’s CGEA spring meeting.*

**PREPARATION FOR/OBSERVATIONS DURING VIRTUAL SESSION** (**Allot 15-20 minutes)**

**1. *What do you wish you would have known ahead of time, if anything, before providing formative feedback to presenters at this year’s CGEA spring meeting?***

***2. How confident were you in providing written feedback? [Probe for past training on giving feedback of this nature]?***

**3. *What information from us would have helped you perform your role?***

**PERCEPTIONS OF FEEDBACK PROCESS (Allot 15-20 minutes)**

*This year we asked MESRE members to observe virtual presentations and provide each speaker with formative feedback.*

**4. *How did you approach providing formative feedback to presenters?***

**5. *What opportunities or challenges did you encounter when providing feedback?***

**6. *What benefits, if any, did you experience in providing feedback?***

***7. What suggestions, if any, do you have for us to improve the evaluation form? [Project form]***

**8. *Should we continue to provide presenters with formative feedback?* *If so,* *what suggestions do you have for us to improve the experience for you? For presenters?***

**OTHER (Allot 5-10 minutes)**

**9. *What else would you like to tell us about this process that we did not already discuss?***

**Structured Guide for Focus Group with Providers of Summative Feedback**

**After the Regional CGEA Conference**

**Number of Participants:**  **Length of Focus Group (Min:Sec):** :

**INTRODUCTION (Allot 3-5 minutes)**

*Thank you for taking the time to meet with us. Our overall goal during this focus group is to learn how the Medical Education Scholarship, Research and Evaluation section – also known as MESRE – can better support the professional development and careers of health professions educators and scholars. We are especially interested in learning more about your experience while providing presenters with summative feedback at this year’s CGEA meeting. We will use your observations to improve how we assist those who provide feedback to others who present educational scholarship at medical education conferences. I would like to record our discussion. This will let me focus on what you say rather than take a lot of notes. Your name will not appear on the minutes of our discussion or my notes. Please use the poll to let me know if recording this session is okay with you [launch poll – record or don’t record based on vote].*

*As we’ve all become more familiar with Zoom, I ask that you turn your camera on so we can see each other during the conversation. I would appreciate equal participation - through dialogue or Zoom features - as everyone’s experience and opinions matter to us. [Display how to access chat and participation icons in Zoom].*

*Let’s begin with briefly introducing ourselves by giving our name, current role, and institution. Then, I’ll ask about your experience at this year’s CGEA spring meeting.*

**PREPARATION FOR/OBSERVATIONS DURING VIRTUAL SESSION** (**Allot 15-20 minutes)**

**1. *What do you wish you would have known ahead of time, if anything, before providing summative feedback to presenters at this year’s CGEA spring meeting?***

**2. *How confident were you in providing feedback? [Probe for past training on giving feedback of this nature]?***

**3. *What information from us would have helped you perform your role?***

**PERCEPTIONS OF FEEDBACK PROCESS (Allot 15-20 minutes)**

*This year we asked MESRE members to observe virtual presentations and provide each speaker with summative feedback.*

**4. *How did you approach providing summative feedback to presenters?***

**5. *What opportunities or challenges did you encounter when providing feedback?***

**6. *What benefits, if any, did you experience in providing feedback?***

***7. What suggestions, if any, do you have for us to improve the rubric? [Project rubric]***

**8. *Should we continue to provide presenters with summative feedback?* *If so,* *what suggestions do you have for us to improve the experience for you? For presenters?***

**OTHER (Allot 5-10 minutes)**

**9. *What else would you like to tell us about this process that we did not already discuss?***
