# Supplementary material for: A Systematic Approach to Provide Feedback to Presenters at Virtual and Face-to-Face Professional Meetings
Source: MedEdPORTAL. 2022 Dec 16;18:11288. doi: 10.15766/mep_2374-8265.11288 (PMC9755373; doi:10.15766/mep_2374-8265.11288)
Supplement: Supplementary file 1 — Meeting Organizer Checklist.docxEmail to Presenters (Before Conference).docxSummative Assessment Forms.docFormative Assessment Form.docxEmail to Assessors (Before Conference).docxEmail to Presenters (After Conference).docxEmail to Assessors (After Conference).docxFocus Group Guides.docx [file mep_2374-8265.11288-s001.zip › Educational Summary Report_11288.pdf]

MedEdPORTAL® | The AAMC Journal of Teaching and Learning Resources

Original Publication

Open Access

A Systematic Approach to Provide Feedback to Presenters at Virtual and Face-to-Face Professional Meetings

S. Beth Bierer, PhD*, Anna Cianciolo, PhD, Heeyoung Han, PhD, Janice Hanson, PhD

*Corresponding author: biererb@ccf.org

Abstract

Introduction: To promote their personal and professional growth, medical educators need practical, actionable feedback on their scholarship, as well as guidance for documenting their scholarship in educator portfolios. We offer a framework and resources to provide formative and summative feedback to faculty, administrators, and/or learners delivering an oral presentation at a face-to-face or virtual health professions education meeting. **Methods:** In 2014, the leadership of the Central Group on Educational Affairs (CGEA) meeting planning committee developed and piloted a process to provide individuals with formative and summative feedback on their oral CGEA research presentations at face-to-face meetings and create a transparent process for determining the Best Presentation Award. The feedback process was implemented for 7 years until revised in 2021 for the CGEA's first virtual meeting. Past and present meeting organizers conducted four focus groups in 2021 with presenters and peer reviewers via Zoom. Transcripts were analyzed for major themes using conventional content analysis. **Results:** To date, 102 presentation assessments have been conducted, including formative and summative assessments. Sixty-two volunteer assessors have participated, 19 (31%) of whom served for more than 2 years. Focus groups identified best practices and suggestions to improve the feedback process. **Discussion:** This resource offers a feasible, systematic process to provide individuals with formative feedback on presentations at professional conferences, promote a community of practice for personal and professional development, and create a transparent process for determining a Best Presentation Award. Participants valued providing and receiving feedback and recommended implementation at other professional meetings.

Keywords
Faculty Development, Feedback, Publishing/Scholarship

Educational Objectives

By the end of this activity, learners will be able to:

1. Describe the value of providing presenters with formative and summative feedback to support their professional development.
2. Identify criteria to evaluate the quality of research presentations in health professions education.
3. Consider assessment forms to provide presenters with formative and summative feedback.
4. Implement a process to support the development of presenters at professional meetings and build capacity for educational scholarship.
5. Identify one approach to foster a community of practice with education scholars who attend professional meetings.

Citation:
Bierer SB, Cianciolo A, Han H, Hanson J. A systematic approach to provide feedback to presenters at virtual and face-to-face professional meetings. *MedEdPORTAL*. 2022;18:11288.
https://doi.org/10.15766/mep_2374-8265.11288

Introduction

Scholarship in health professions education (HPE) has developed substantially in recent decades.[1] Whereas at one time scholarly dissemination (i.e., presentations and publications) about HPE focused on sharing examples of teaching activities and learners' satisfaction, more recent dissemination reflects a wide range of teaching and inquiry methods. To disseminate their work, education scholars must apply rigorous inquiry, use theoretical and conceptual frameworks to develop and communicate their work, assess meaningful learning outcomes,[2-4] and provide inclusive language.[5] Understanding how to plan, carry out, and disseminate the results of scholarship in HPE requires access to many opportunities to learn and practice scholarly activities.

Academic health centers and professional organizations have developed various approaches to promote scholarship in HPE and build the capacity of learners and faculty to participate,

Copyright © 2022 Bierer et al. This is an open-access publication distributed under the terms of the Creative Commons Attribution license.

including creating education units,[6-9] academies of educators,[10] educational scholars programs, and other faculty development opportunities.[11-14] Some academic health centers have facilitated regular meetings of education scholars to discuss works in progress[15] or cross-campus research collaborations for education scholarship.[16]

Academic health centers and professional organizations also promote education scholarship by hosting scholarly dissemination events, such as meetings or conferences, where education scholars present and discuss their work.[17] Through collegial interactions with peers, these events provide a developmental opportunity to build scholars' capacity for high-quality scholarship and presentation skills while facilitating their professional identity development as members of a community of practice.[18] A community of practice is a relational network in which members observe, learn, and participate in a practice, first at the periphery of the group and then with increasing engagement. In this resource, *community* comprises education scholars, and their *practice* is the work of doing and presenting education scholarship.[19,20] Supporting members' development promotes self-efficacy, engages members as learners and peers, and furthers the experience of the community. A community of practice in HPE scholarship provides opportunities for experienced scholars to give back to the community and for junior scholars to mature in their ability to do and share scholarly work. Within a community of practice, formative and summative feedback provides a time-tested approach to teaching and learning and for promoting the engagement of members with varying levels of experience.

The target audience for this assessment resource includes meeting planners who want to build a systematic process to offer feedback on presentations delivered at in-person or virtual scholarly dissemination events. Our resource provides a structure to support faculty scholarly development via professional meetings or conferences that include presentations about education scholarship, within a community of medical educators who learn and promote education scholarship. Furthermore, this resource includes practical tools for those who convene such events.

While there are tools in *MedEdPORTAL* for planning and convening a symposium of education research and scholarship,[17] we are not aware of a publication in *MedEdPORTAL* or elsewhere that features tools for providing feedback on oral presentations as an approach to learning about scholarly dissemination and building a community of practice. Our resource provides a practical, feasible way to develop skills for high-quality scholarship in the context of a meeting and the presentations that occur. We also do not know of other work that has followed up with recipients and providers of feedback on education scholarship presentations about how this process contributes to their professional growth and experience of community. With this assessment resource, we aim to address that gap.

Methods

This assessment resource offered a framework for providing feedback to faculty, administrators, and/or learners delivering an oral presentation at a face-to-face or virtual HPE meeting. Our intent, as past and present regional chairs of the Medical Education, Scholarship, Research and Evaluation (MESRE) section of the Central Group on Educational Affairs (CGEA), was to offer presenters—particularly those with less experience at medical education conferences—specific guidance on how to prepare and deliver an effective oral presentation and provide them with formative feedback that they could use to improve their research and their presentation skills. Additionally, we sought a fair and defensible summative assessment approach in which to select a presenter to receive an award for the best oral presentation.

Implementation at Face-to-Face Professional Meetings

In 2014, the CGEA's steering committee decided to provide presenters with summative and formative feedback after they had attended the CGEA regional conference. The CGEA MESRE chair (Beth Bierer), based on experience with programmatic assessment, designed separate forms to provide presenters with summative and formative feedback given the different purpose of each type of feedback. The summative assessment form focused on having assessors use numeric rating scales to rate the content and delivery of oral presentations to determine recipients for the Best Presentation Award at the CGEA conference. The formative assessment form asked assessors to provide individualized, narrative feedback to each presenter, with the goal of offering encouragement and advice in order to advance scholarship. Both assessment forms were based on input from the CGEA steering committee, as a literature review did not provide guidance. The two feedback forms were piloted at the face-to-face 2014 CGEA conference. During subsequent years, MESRE section chairs continued to provide presenters with summative and formative feedback given the initial success of this feedback process.

Pilot for Virtual Professional Meetings

Informed by our experience as past and present regional MESRE chairs, we met on several occasions in the fall of 2020 to adapt the existing assessment forms and feedback protocol in anticipation of the 2021 CGEA spring conference going

Copyright © 2022 Bierer et al. This is an open-access publication distributed under the terms of the Creative Commons Attribution license.

virtual in response to the COVID-19 pandemic. The summative assessment form was revised to reflect unique aspects of delivering a presentation at a virtual meeting (e.g., audio quality, appropriate lighting). The formative assessment form and feedback protocol (i.e., distributing assessment forms to presenters prior to the conference with instructions, recruiting assessors, determining the Best Presentation Award, distributing feedback to presenters and assessors, etc.) remained the same. The regional MESRE chair (Heeyoung Han) piloted the updated feedback process at the 2021 CGEA virtual conference, and the current MESRE chair (Janice Hanson) repeated the updated protocol at the 2022 CGEA virtual conference.

Recommended Feedback Protocol

Based on our years of experience with this feedback protocol in face-to-face and virtual contexts, we developed a detailed checklist and timeline for those wishing to replicate our feedback approach (Appendix A) at other scholarly dissemination events. The summary below provides an overview of this process as context for the appendices.

- Before the event:
  - Generate a list of the complete names and email addresses of presenters selected to give an oral presentation.
  - Send an email to each presenter offering guidance on how to prepare an effective oral presentation (Appendix B). Include examples of the blank summative and formative assessment forms (Appendices C and D).
  - Recruit assessors with expertise in medical education who plan to attend the event. Provide a task description and expectations for each role (formative and summative). After identifying assessors, ask them to specify their preference for providing formative or summative feedback.
  - Develop a schedule where two summative assessors and one formative assessor are assigned to provide feedback for four to five presentations. Pay attention to potential conflicts of interest (e.g., summative assessors are not from the same institution as the presenters) and scheduling (e.g., assessor is giving a presentation at the same time as the assigned session). If feasible, distribute the schedule to assessors in advance, and ask them to specify the set of presentations on which they would prefer to provide feedback.
  - Distribute a final schedule, links to electronic assessment forms, assessors' specific presentation assignments, and conference abstracts for assigned presentation assignments to assessors (Appendix E).
  - If feasible, recruit session moderators who can introduce speakers and solicit questions/monitor the chat if the meeting is virtual. The meeting organizer could also ask the summative assessor to serve as a session moderator.
  - Send a reminder to assessors and moderators before the event.
- During the event:
  - If a face-to-face meeting, have meeting organizers verify that moderators and assessors arrive at the correct locations. If a virtual meeting, have someone qualified to serve as an assessor or moderator on standby in case an assessor or moderator experiences technical problems and cannot access the assigned session.
  - Have the feedback organizer—in our case, it was the MESRE chair—monitor for timely submission of assessments.
- After the event:
  - Approximately 2-3 days after the event, compile and aggregate the summative assessment forms, and determine the recipient of the Best Presentation Award. For our conference, the meeting organizer averaged the ratings of the two summative assessments for each presenter and ranked the presenters based on their ratings to identify the recipient of the Best Presentation Award. Additionally, compile all formative and summative assessment forms for each presenter, and create PDF files to distribute to individual presenters.
  - Distribute a personalized email (Appendix F) to each presenter that (1) announces the recipient of the Best Presentation Award and (2) includes the aggregated feedback document as an attachment.
  - Distribute a personalized email (Appendix G) to each group of assessors assigned to provide formative and summative feedback to a specific group of presenters. This email should announce the recipient of the Best Presentation Award, include collated feedback to presenters, present observations on the feedback process, and solicit feedback from assessors about the process.

Program Evaluation

The conceptual framework underlying this curricular resource was that of a community of practice.[18] We thought of the members of a professional organization involving education research and scholarship as a community of scholars who could support learning the abilities for conducting and presenting education work. Therefore, we explored community development

Copyright © 2022 Bierer et al. This is an open-access publication distributed under the terms of the Creative Commons Attribution license.

and engagement. Consequently, our data collection focused on presenter and assessor involvement and perceptions of the usefulness of providing and receiving feedback. We used separate focus groups for assessors and presenters (Appendix H) to obtain stakeholders' perceptions about the feedback process, after receiving approval from the Springfield Committee for Research Involving Human Subjects and the Cleveland Clinic's Office of Institutional Research. Following the 2021 CGEA spring meeting, the CGEA MESRE chair (Heeyoung Han) sent a separate email invitation to presenters and assessors, inviting them to join one of four focus groups via Zoom. Focus groups were digitally recorded and transcribed via Zoom, supported by notes compiled after each focus group. All authors independently read transcripts and notes and then met as a group, via Zoom, to discuss major themes and action items for future implementation of the feedback protocol.

Results

Participants

Over the last 8 years, 102 presentation assessments were conducted, including formative and summative assessments (Table 1), except in 2020, when the CGEA spring meeting was canceled due to the COVID-19 pandemic. Sixty-two volunteers participated as assessors, 19 (31%) of whom served as assessors for more than 2 years.

We conducted four focus group interviews with eight participants (four assessors and four presenters) in June 2021 to understand participants' perspectives regarding strengths and recommended improvements. Below, we summarize key points from these focus groups, with representative comments presented in Table 2.

Benefits

Appreciation of formative and narrative feedback as guidance for further scholarship: The presenters found the formative and narrative feedback helpful, instructive, thorough, and useful for their presentations as well as for future scholarship. They shared that the formative feedback included actionable advice to improve their research and motivated them to continue their scholarship, such as preparing a journal article submission.

Appreciation of narrative feedback quality: Presenters reported that feedback quality differed from what they received at other events, as it came from experts familiar with their topics. This finding was consistent with our recruitment practice, whereby we tried to align the presentations with each assessor's expertise. The finding also aligned with assessors' self-reported confidence as feedback providers. Assessors said they had baseline knowledge of what average presentations were like at CGEA meetings and were comfortable providing feedback based on their expertise.

Symbolic benefits for assessors: Assessors also reported benefits, saying that scholarly service could support career development, especially for those on a research track. However, assessors did not always describe direct, practical benefits for their jobs but, rather, symbolic benefits with respect to altruism. For example, some assessors reported working at institutions that prioritized clinical research over educational research, where there was no job-related reward for providing feedback on education scholarship, yet they volunteered because they wanted to help presenters appreciate their own work and to provide actionable guidance for improvement.

Rigorous process facilitated community building: Participants appreciated our feedback process as a well-designed, rigorous system that facilitated scholarly community building. Meaningful and constructive feedback facilitated assessors' and presenters' engagement and participation in the event by showing value in community development. Assessors were inspired to learn about various projects happening at other schools, and due to their feedback assignment, they were engaged in the presentations and discussions.

Areas for Improvement

Orientation and more reminders: Although we provided both assessors and presenters with requisite information, including assessment criteria, forms, abstracts, and actual presentation slides via emails, some of them, especially newcomers and presenters, did not seem to pay attention to the materials. Some assessors, especially formative assessors, found it helpful to have abstracts in advance, whereas others asked for all materials and

Table 1. Number of Formative and Summative Assessments Distributed to Presenters by Conference Year

Assessment Type	2014	2015	2016	2017	2018	2019	2020[a]	2021	Total
Formative	5	5	5	5	5	5	NA	4	34
Summative	10	10	10	10	10	10	NA	8	68
Total	15	15	15	15	15	15	NA	12	102

Abbreviation: NA, not applicable.
[a]The 2020 Central Group on Educational Affairs spring conference was canceled due to COVID-19.

Copyright © 2022 Bierer et al. This is an open-access publication distributed under the terms of the Creative Commons Attribution license.

Table 2. Focus Group Themes With Supporting Statements From Assessors and Presenters

Themes and Subthemes	Supporting Statements From Focus Groups
Benefits	
Appreciation of formative and narrative feedback as guidance for further scholarship	"It kind of really is just a great opportunity to see some of this work that people are doing and support them and feel like we are, you know, building this community." (Assessor)<br>"These presentations are often a stepping stone on the way to a publication, and so you want to be able to give them some feedback that you think will help them as they're moving to the next step." (Assessor)
Appreciation of narrative feedback quality	"The descriptive response is unique to the presenter." (Presenter)<br>"The feedback was very helpful, instructive, and thorough." (Presenter)<br>"There were a few comments I appreciated… [described specifics about graphic]… that gave me something to work on." (Presenter)
Symbolic benefits for assessors	"I've been doing these things out of the goodness of my heart, because I get no academic credit for it. And that gave me great intrinsic satisfaction that I enjoy doing it and I enjoyed the relationships I've created as a result." (Assessor)<br>"One of the benefits of… providing feedback or being involved in reviewing in some way [is] you pay a lot more attention." (Assessor)
Rigorous process facilitated community building	"The value [of this feedback process], compared to other conferences, is that you're getting feedback from people who've been around for a while that know how to do this, instead of relying on the audience to fill out evaluation forms that most commonly don't get completed and are a mixed bag at best…. We're actually trying to help each other and build a community of practice and nurture new generations…. I think this is a great way to do that." (Assessor)<br>"It was really inspiring and fun to hear about what these folks are working on and I really enjoy going to these abstracts [presentations]. It really is just a great opportunity to see some of this work that people are doing and support them, and feel like we are building this Community together, so that's what I get out of it." (Assessor)
Areas for improvement	
Orientation and more reminders	"I would make that really clear and advertise that to presenters so they know how they're being judged when they present." (Assessor)
Clarifying evaluation focus	"[Provide] some pointed feedback that helps them to go from one level to the next, but at the same time being encouraging and not discouraging of what was really interesting." (Assessor)<br>"Some [aren't] English native speakers…. Your presentation skills become better and better when you do more and more presentations, but the content [is] very solid so that's why I'm more focused on their content." (Assessor)
Time management and mental break	"Having the moderator role separate from the summative assessor role does really help…. I think this was much easier because you have time in between." (Assessor)
Other suggestions	
Assessor development	"[I] wonder if some of the more junior reviewers might appreciate being paired up with a more senior reviewer or having a training session…. That might help them… as a junior researcher, to have a chance to review with somebody else and kind of get the idea of how that [can be done]." (Assessor)<br>"Feedback should be shared with formative assessors, just like journal reviews, you know, so people can… learn from each other." (Assessor)
Assessor recognition	"I think for most people that [thank-you note to assessor and copy department chair] is probably very helpful." (Assessor)
Revisiting the summative assessment items	"The presenters weren't visible [during virtual presentations], and so eye contact was irrelevant." (Assessor)
Sharing this program with other conference groups	"I'm sure you guys are aware of the way it used to be in the old days…. It was a cutthroat… to go to present and most often people tried to make themselves look good by criticizing the presenters in the room…. We're actually trying to help each other and build a community of practice and nurture a generation… and I think this is a great way to do that." (Assessor)<br>"I like the idea of sharing this with other people… because I think it's valuable." (Assessor)

information ahead of time even though we had already provided the materials and information.

Clarifying evaluation focus: Assessors expressed uncertainty regarding how to focus/balance presentation skill versus content in their assessments. One participant mentioned focusing on both presentation skills and content, but another assessor, who was not a native English speaker, shared that it was hard to judge presentation skills.

Time management and mental break: Assessors shared that multitasking and time management during presentations were challenging, especially for formative assessment. Some assessors felt that formative feedback would be too brief if they did not prepare feedback drafts in advance. Given the time limit during presentations, some assessors suggested having two alternating formative assessors per session to provide a mental break between presentations.

In earlier years of our feedback process, one of the two summative assessors also served as the session moderator. In 2021, the virtual format allowed us to separate these roles. Assessors appreciated this change, given the issues with time management.

Other Suggestions

Assessor development: Securing assessors able to provide valuable feedback required a pipeline approach. One of the ideas participants shared was to use this feedback process for assessor development, especially for junior community members who

Copyright © 2022 Bierer et al. This is an open-access publication distributed under the terms of the Creative Commons Attribution license.

might not have mentors at their institutions. For example, senior and junior community members could be paired as assessors, producing a collaborative review. Alternatively, assessors could invite their trainees. Presenters suggested getting involved in assessing, as seeing a variety of quality among presentations could provide the best way to learn presentation skills and develop a professional identity. Sharing formative reviews among formative assessors could help assessors learn from each other regarding effective presentation skills.

Assessor recognition: Assessors recommended sending thank-you notes and publishing assessors' names in the CGEA program to recognize their service.

Revisiting the summative assessment items: Participants suggested revising the behavioral anchors in the summative feedback form as they might not adapt to each presentation/context (e.g., eye contact, institutional review board). For example, virtual presentations were prerecorded with only slides and audio, making it impossible to assess eye contact during the presentation.

Sharing this program with other conference groups: Participants thought other conferences should adopt this peer feedback process.

Discussion

We set out to create a process for providing formative and summative feedback and selecting presenters for awards at a meeting of medical educators. We met these goals and learned that the process also facilitated the development of a community of practice for education scholars in our professional organization. We developed this assessment resource to engage experienced scholars in providing a forum to share education scholarship in a way that would promote skill development in a community of practice. As illustrated by the number of reoccurring assessors and the constant influx of new assessors in our process, developing a feedback process and a set of assessment resources (i.e., the appendices) has supported the sustainability of the process among successive MESRE chairs and has helped our professional organization mentor the next generation of education scholars.

Our assessment resource can be appreciated by institutions and those in charge of professional meetings. The detailed information we provide offers clear guidelines for assessing scholarly presentations and an approach for sustaining a community of scholars. Through the feedback process we created, experienced community members share their wisdom with junior or early career community members. These assessors see the program's value and are willing to help others. This process mobilizes a community of scholarly educators based on their needs, expertise, and commitment to the profession. It also works as a succession-planning process for the next generation. Another value of this assessment resource is to support participants' professional development. Presenters use actionable feedback from experienced community members to advance their scholarly output, such as publication. Sharing these assessment resources and a detailed description of the feedback process can enable leaders in other organizations to replicate and adapt this approach, thereby facilitating a community of practice in other groups of education scholars.

This assessment resource requires careful planning and central oversight for successful implementation. However, we believe the benefits for both presenters and peer reviewers, as summarized in Table 2, outweigh the time commitment for meeting planners. We had the benefit of recruiting assessors from established scholars and sharing our list of assessors with future meeting organizers. Others may need to develop a pool of qualified assessors. Over the years, we had only one incident of a presenter taking feedback personally. In response, we adapted the scale of the summative assessment form from 0-3 to 1-4 to ensure presenters would not receive artificially low scores. Though we implemented this feedback approach at regional medical education conferences, we believe it provides a transferable, feasible model for institutional symposia or specialty meetings. Finally, we did not focus our program evaluation on psychometric properties of the assessment forms or obtained data (i.e., reliability). Instead, we examined the intended and unintended outcomes after implementation. Future research should explore the extent to which this resource fosters a community of practice and promotes personal and professional development. Specific markers of a community of practice could form a foundation for evaluation of the application of this resource in a new setting. Program leaders can assess the engagement of assessors of presentations over time, the development of new mentoring relationships in the organization, the value of feedback to presenters as they move their research forward, and the numbers of presentations moving to publication as outcomes of the skill-building feedback process.

Appendices

A. Meeting Organizer Checklist.docx

B. Email to Presenters (Before Conference).docx

Copyright © 2022 Bierer et al. This is an open-access publication distributed under the terms of the Creative Commons Attribution license.

C. Summative Assessment Forms.doc

D. Formative Assessment Form.docx

E. Email to Assessors (Before Conference).docx

F. Email to Presenters (After Conference).docx

G. Email to Assessors (After Conference).docx

H. Focus Group Guides.docx

All appendices are peer reviewed as integral parts of the Original Publication.

S. Beth Bierer, PhD: Professor, Department of Medicine, and Director, Assessment and Evaluation, Cleveland Clinic Lerner College of Medicine of Case Western Reserve University; ORCID: https://orcid.org/0000-0002-7952-8822

Anna Cianciolo, PhD: Associate Professor, Department of Medical Education, Southern Illinois University School of Medicine; ORCID: https://orcid.org/0000-0001-5948-9304

Heeyoung Han, PhD: Associate Professor, Department of Medical Education, Southern Illinois University School of Medicine; ORCID: https://orcid.org/0000-0002-7286-2473

Janice Hanson, PhD: Professor, Department of Medicine, Washington University School of Medicine in St. Louis; ORCID: https://orcid.org/0000-0001-7051-8225

Disclosures
None to report.

Funding/Support
None to report.

Ethical Approval
The Springfield Committee for Research Involving Human Subjects and the Cleveland Clinic's Office of Institutional Research deemed further review of this project not necessary.

References

1. Irby DM, O'Sullivan PS. Developing and rewarding teachers as educators and scholars: remarkable progress and daunting challenges. *Med Educ*. 2018;52(1):58-67. https://doi.org/10.1111/medu.13379
2. West DC, Miller KH, Artino AR Jr. Foreword: characteristics of RIME papers that make the cut. *Acad Med*. 2016;91(11)(suppl):Si-Siii. https://doi.org/10.1097/ACM.0000000000001379
3. Abu-Rish E, Kim S, Choe L, et al. Current trends in interprofessional education of health sciences students: a literature review. *J Interprof Care*. 2012;26(6):444-451. https://doi.org/10.3109/13561820.2012.715604
4. Varpio L, Paradis E, Uijtdehaage S, Young M. The distinctions between theory, theoretical framework, and conceptual framework. *Acad Med*. 2020;95(7):989-994. https://doi.org/10.1097/ACM.0000000000003075
5. *Advancing Health Equity: A Guide to Language, Narrative and Concepts.* American Medical Association/Association of American Medical Colleges; 2021. Accessed August 21, 2022. https://www.ama-assn.org/system/files/ama-aamc-equity-guide.pdf
6. Cofrancesco J Jr, Barone MA, Serwint JR, Goldstein M, Westman M, Lipsett PA. Development and implementation of a school-wide Institute for Excellence in Education to enable educational scholarship by medical school faculty. *Teach Learn Med*. 2018; 30(1):103-111. https://doi.org/10.1080/10401334.2017.1325741
7. Humphrey-Murto S, O'Brien B, Irby DM, et al. 14 years later: a follow-up case-study analysis of 8 health professions education scholarship units. *Acad Med*. 2020;95(4):629-636. https://doi.org/10.1097/ACM.0000000000003095
8. Klein M, Real FJ, Kachelmeyer A, et al. Development of a pediatric education research unit. *J Pediatr*. 2020;220:4-6.e1. https://doi.org/10.1016/j.jpeds.2019.12.007
9. Nicklas D, Treitz M, Daniels S, Hanson J. Developing a Center for Education Research and Scholarship in a department of pediatrics [version 1]. *MedEdPublish*. 2020;9:181. https://doi.org/10.15694/mep.2020.000181.1
10. Uijtdehaage S, Ho MJ, Harvey E, Dorris CS, Huggett KN. Academies in health professions education: a scoping review. *Acad Med*. 2021;96(10):1476-1483. https://doi.org/10.1097/ACM.0000000000004161
11. Baldwin CD, Gusic ME, Chandran L. The impact of a national faculty development program embedded within an academic professional organization. *Acad Med*. 2017;92(8):1105-1113. https://doi.org/10.1097/ACM.0000000000001496
12. Cottingham AH, Sachs GA, Frankel RM. ASPIRE: a program for developing clinician educators' scholarship, advancement, and sense of community. *J Gen Intern Med*. 2022;37(8):1953-1962. https://doi.org/10.1007/s11606-021-07243-0
13. Gruppen LD, Frohna AZ, Anderson RM, Lowe KD. Faculty development for educational leadership and scholarship. *Acad Med*. 2003;78(2):137-141. https://doi.org/10.1097/00001888-200302000-00007
14. Jerardi KE, Mogilner L, Turner T, Chandran L, Baldwin CD, Klein M. Investment in faculty as educational scholars: outcomes from the national Educational Scholars Program. *J Pediatr*. 2016;171:4-5.e1. https://doi.org/10.1016/j.jpeds.2015.12.052
15. Schwengel DA, Toy S. Innovation in education research: creation of an education research core. *Anesth Analg*. 2019;129(2): 520-525. https://doi.org/10.1213/ANE.0000000000003971
16. Peterson WJ, Santen SA, House JB, et al. Increasing education research productivity: a network analysis. *West J Emerg Med*.

Copyright © 2022 Bierer et al. This is an open-access publication distributed under the terms of the Creative Commons Attribution license.

2019;21(1):163-168.
https://doi.org/10.5811/westjem.2019.12.44512

17. Wolfe AD, Bhalala U, Marshall V. Building a local research symposium: the crossroads of scholarship, education, and faculty development. *MedEdPORTAL*. 2020;16:11048. https://doi.org/10.15766/mep_2374-8265.11048
18. Cruess RL, Cruess SR, Steinert Y. Medicine as a community of practice: implications for medical education. *Acad Med*. 2018;93(2):185-191. https://doi.org/10.1097/ACM.0000000000001826
19. Omidvar O, Kislov R. The evolution of the communities of practice approach: toward knowledgeability in a landscape of practice—an interview with Etienne Wenger-Trayner. *J Manag Inq*. 2014;23(3):266-275. https://doi.org/10.1177/1056492613505908
20. Hodson N. Landscapes of practice in medical education. *Med Educ*. 2020:54(6):504-509. https://doi.org/10.1111/medu.14061

Received: May 20, 2022
Accepted: September 6, 2022
Published: December 16, 2022

Copyright © 2022 Bierer et al. This is an open-access publication distributed under the terms of the Creative Commons Attribution license.
