# Supplementary material for: A Systematic Approach to Provide Feedback to Presenters at Virtual and Face-to-Face Professional Meetings
Source: MedEdPORTAL. 2022 Dec 16;18:11288. doi: 10.15766/mep_2374-8265.11288 (PMC9755373; doi:10.15766/mep_2374-8265.11288)
Supplement: Supplementary file 1 — Meeting Organizer Checklist.docxEmail to Presenters (Before Conference).docxSummative Assessment Forms.docFormative Assessment Form.docxEmail to Assessors (Before Conference).docxEmail to Presenters (After Conference).docxEmail to Assessors (After Conference).docxFocus Group Guides.docx [file mep_2374-8265.11288-s001.zip › C. Summative Assessment Forms.doc]

Oral Presentation – Summative Feedback Form for Face-to-Face Presentation (2019 Version)

Presentation Title:	Session#:

Presenter:	Date:

| Introduction | 1   - Doesn’t orient the audience to topic of presentation - Doesn’t give audience sense of what to expect in presentation | 2   - Some structure but does describe what follows - Overly detailed or incomplete introduction of topic | 3   - Presents overall topic - Clearly states the organization of the presentation | 4   - Presents topic clearly - Connects with audience (poses questions, acknowledges audience’s interests/goals) |
| --- | --- | --- | --- | --- |
| Problem/  Goals | 1   - Lacks problem statement and/or goals | 2   - Problem/goal not clear - Evidence presented does not support the problem/goals | 3   - Well posed problem statement - Provides some evidence to justify project | 4   - Stated problem clearly - Explains why research project was undertaken |
| Methods | 1   - Description of methods is incoherent or missing - Procedures discussed only briefly or abstractly - Design is confusing/incomplete | 2   - Describes some methods - Unclear data collection procedures - Data collection procedures aligned to questions | 3   - Describes methods but relevance to topic not apparent - Data collection procedures aligned to questions | 4   - Describes methods clearly - Clear data collection - Methods support questions and project goals |
| Project  Significance | 1   - Not discussed | 2   - Superficial statement of impact - Only hints at potential impact | 3   - Significance discussed - States potential impact | 4   - Significance discussed clearly - Potential impact on research or practice is explained |
| Challenges/  Limitations | 1   - Does not address project challenges and limitations | 2   - Addresses some challenges and limitations | 3   - Identifies the significant challenges and limitations of the project - Does not discuss implications | 4   - Identifies the significant challenges and limitations of project - Describes the implications for applying or extending the findings |
| Audiovisual | 1   - Audiovisual aids do not complement the information presented | 2   - Too few or too many aids - Difficult to understand aids - Doesn’t refer to or explain aids | 3   - Uses appropriate and easily understood audiovisual aids | 4   - Uses appropriate and easily understood audiovisual aids that enhance the quality of the presentation |
| Delivery | 1   - Poor eye contact - Volume too low or loud; poor enunciation. - Relied heavily on audiovisuals - Not timed appropriately - Uses non-inclusive language (e.g., gendered pronouns, insensitivity terminology) | 2   - Inconsistent with eye  contact, volume, and  enunciation - Looked at audiovisuals often to stay on track - Acceptable use of time - Language is neither inclusive nor non-inclusive | 3   - Good eye contact - No distracting gestures - Usually had appropriate  volume and enunciation - Good use of time   Some use of inclusive language (e.g., gender-neutral pronouns, person-centered terminology | 4   - Excellent eye contact - No distracting gestures - Appropriate volume - Clear enunciation - Excellent time management - Intentional use of inclusive language |

| Overall  Impressions | 1   - Topic does not address perceived need/challenge - Limited appeal/impact of project to other schools | 2   - Topic explores perceived need/challenge - Speaker demonstrates some   knowledge of topic   - Limited appeal/impact of project to other schools | 3   - Important/timely topic - Presentation has “So what?” component - Speaker is clearly knowledgeable - Project has clear appeal to other schools | 4   - Important/timely topic - Presentation has “Now what?” component - Speaker is clearly knowledgeable - Project has clear appeal to other schools |
| --- | --- | --- | --- | --- |
| Research  Ethics | Presenter specified obtaining IRB approval in presentation | Yes No Not Applicable | | |

Score: /32

Please write additional comments/observations for presenter:

Oral Presentation – Summative Feedback for Virtual Presentation (2021 Version)

Title of Presentation:	No. of Session:

Presenter:	Date:

| Introduction | 1   - Doesn’t orient the audience to topic of presentation - Doesn’t give audience sense of what to expect in presentation | 2   - Some structure but does describe what follows - Overly detailed or incomplete introduction of topic | 3   - Presents overall topic - Clearly states the organization of the presentation | 4   - Presents topic clearly - Connects with audience (poses questions, acknowledges audience’s interests/goals) |
| --- | --- | --- | --- | --- |
| Problem/  Goals | 1   - Lacks problem statement and/or goals | 2   - Problem/goal not clear - Evidence presented does not support the problem/goals | 3   - Well posed problem statement - Provides some evidence to justify project | 4   - Stated problem clearly - Explains why research project was undertaken |
| Methods | 1   - Description of methods is incoherent or missing - Procedures discussed only briefly or abstractly - Design is confusing/incomplete | 2   - Describes some methods - Unclear data collection procedures - Data collection procedures aligned to questions | 3   - Describes methods but relevance to topic not apparent - Data collection procedures aligned to questions | 4   - Describes methods clearly - Clear data collection - Methods support questions and project goals |
| Project  Significance | 1   - Not discussed | 2   - Superficial statement of impact - Only hints at potential impact | 3   - Significance discussed - States potential impact | 4   - Significance discussed clearly - Potential impact on research or practice is explained |
| Challenges/  Limitations | 1   - Does not address project challenges and limitations | 2   - Addresses some challenges and limitations | 3   - Identifies the significant challenges and limitations of the project - Does not discuss implications | 4   - Identifies the significant challenges and limitations of project - Describes the implications for applying or extending the findings |
| Audiovisual of Presentation/ Slides | 1   - Audiovisual aids do not complement the information presented | 2   - Too few or too many aids - Difficult to understand aids - Doesn’t refer to or explain aids | 3   - Uses appropriate and easily understood audiovisual aids | 4   - Uses appropriate and easily understood audiovisual aids that enhance the quality of the presentation |
| Delivery in the virtual environment | 1   - Poor eye contact. - No eye gaze into the screen/camera - Not putting the camera at the eye level - Volume too low or loud; poor enunciation - Read directly the words on the slides; relied heavily on audiovisuals - Not timed appropriately - Uses non-inclusive language (e.g., gendered pronouns, insensitive terminology | 2   - Inconsistent with eye  contact, eye gaze into screen/camera, volume, and enunciation - No putting the camera at eye level - Sometimes used a conversational tone - Looked at audiovisuals often to stay on track - Acceptable use of time - Language is neither inclusive nor non-inclusive | 3   - Good eye contact - Good eye gaze into the screen/camera, while not putting camera at eye level - No distracting gestures - Usually had appropriate  volume and enunciation - Fairly consistent conversational tone - Good use of time - Some use of inclusive language (e.g., gender-neutral pronouns, person-centered terminology) | 4   - Excellent eye contact - Excellent eye gaze into the screen/camera - Put camera at eye level - No distracting gestures - Appropriate volume - Clear enunciation - Engaging, conversational tone and sense of presence throughout the presentation - Excellent time management - Intentional use of inclusive language |

| Delivery in the virtual environment (technical) | 1   - Presenter’s audio quality is poor - It is hard to hear the presentation | 2   - Presenter’s audio quality is inconsistent | 3   - Presenter’s audio quality is appropriate while echo or feedback may present | 4   - Presenter’s audio quality is clear without echo or feedback (using a head set) |
| --- | --- | --- | --- | --- |
| Overall  Impressions | 1   - Topic does not address perceived need/challenge - Limited appeal/impact of project to other schools | 2   - Topic explores perceived need/challenge - Speaker demonstrates some   knowledge of topic   - Limited appeal/impact of project to other schools | 3   - Important/timely topic - Presentation has “So what?” component - Speaker is clearly knowledgeable - Project has clear appeal to other schools | 4   - Important/timely topic - Presentation has “Now what?” component - Speaker is clearly knowledgeable - Project has clear appeal to other schools |
| Professionalism in virtual environment | 1   - Presenter has poor online appearance that is distracting to the observer (e.g., lighting that obscures speaker’s face, unprofessional clothing, etc.) | 2   - Presenter has ineffective online appearance that is noticeable but not distracting | 3   - Presenter has effective online appearance | 4   - Presenter has adapted appearance for online presentation (e.g., effective use of lighting to highlight face, professional dress that does not clash or blend in with background, etc.) |

| Research  Ethics | Presenter specified obtaining IRB approval in presentation | Yes No Not Applicable |
| --- | --- | --- |

Score:      /40

Please write additional comments/observations for presenter:
